# Ultra-processed food consumption and quality of life: a cross-sectional study in Iranian women

**DOI:** 10.3389/fpubh.2024.1351510

**Published:** 2024-04-11

**Authors:** Dorsa Hosseininasab, Farideh Shiraseb, Niki Bahrampour, Alessandra da Silva, Mohammad Mahdi Hajinasab, Josefina Bressan, Khadijeh Mirzaei

**Affiliations:** ^1^Department of Nutrition, Science and Research Branch, Islamic Azad University, Tehran, Iran; ^2^Department of Community Nutrition, School of Nutritional Sciences and Dietetics, Tehran University of Medical Sciences (TUMS), Tehran, Iran; ^3^Laboratory of Energy Metabolism and Body Composition, Department of Nutrition and Health, Universidade Federal de Viçosa, Viçosa, Brazil; ^4^Department of Nutrition, Electronic Health and Statistics Surveillance Research Center, Science and Research Branch, Islamic Azad University, Tehran, Iran

**Keywords:** ultra-processed food, quality of life, obesity, overweight, women

## Abstract

**Background:**

Ultra-processed foods (UPFs) have been associated with a higher intake of added sugars, sodium, and unhealthy fats; however, the relationship between UPFs and quality of life (QoL) is not well understood.

**Methods:**

The present cross-sectional study included 193 Iranian women aged 18–48 years with a body mass index (BMI) of ≥25 kg/m^2^. UPFs were identified using the NOVA classification. QoL was evaluated using the SF-36 questionnaire. Anthropometric measurements and body composition were assessed using an appropriate method.

**Results:**

The mean BMI and fat-free mass (FFM) of the subjects were 30.90 kg/m^2^ and 46.69 kg, respectively. At first, a significant difference was observed in the height of women across tertiles of UPF consumption. The mean score of the total QoL scale was 66.90. Women who were in the tertile 3 of UPFs intake had 23.59 units lower the scale of limitation in physical capabilities and activity (score of role-physical) (*β* = −23.59, 95% CI: −37.77–9.40, *p* = 0.001). Among those with the highest adherence to UPF intake, there was an 8.76 unit reduction in addressing feelings of energy and fatigue (vitality domain) in model 2 (*β* = −8.76, 95% CI: −16.42–1.11, *p* = 0.02). Finally, a reduction of 15.78 units was observed in the mental health scale, specifically in the mental states of anxiety and depression, among participants in the third tertile of UPF intake (*β* = −15.78, 95% CI: −24.11–7.45, *p* < 0.001).

**Conclusion:**

Increased UPF consumption was associated with lower QoL in Iranian women. Further studies are needed to confirm these findings and develop effective strategies to promote healthy food choices.

## Introduction

Finding ways to increase the quality of life (QoL) has always been one of the concerns of scientists. QoL is usually categorized into five dimensions: physical, material, social, and emotional wellbeing, and development and activity (1). Two individuals with the same health condition can have different QoLs based on their expectations and resilience toward health or illness, socioeconomic condition, age, and social support (2–4). Studies have considered QoL as a means to assess healthcare options, particularly for individuals with chronic/disabling diseases (5, 6). Rathnayake et al. indicated that women tend to express lower QoL (compared to men) (7). Additionally, differences in QoL scores have been identified between obese and non-obese women (8). Nutrition and eating habits may be related to individuals’ QoL and can significantly impact it (9); therefore, diverse eating indices have been known to evaluate the quality of a person’s nutrition.

Consumption of ultra-processed foods (UPFs) is increasing rapidly worldwide. According to the Nationwide Food Surveys, UPFs account for 25–60% of total daily energy consumption (10). Based on the NOVA classification system, UPFs are classified as foods made up entirely/predominantly from unhealthy components consisting of greater levels of total fat, saturated fat, added sugar, high-calorie content, salt, and lower fiber and vitamin content (11). Several studies displayed that consumption of UPFs is related to unfavorable health outcomes, including obesity (12–14). Our recent study reported an association between cardiometabolic risk factors and the consumption of UPFs (15).

Although several studies have assessed the relationship between eating habits and health outcomes, such as cardiovascular diseases, dementia, and mortality, studies evaluating the connection between eating habits and QoL are limited. Therefore, we intended to evaluate the relationship between UPF consumption and QoL among obese and overweight Iranian women.

## Methods

### Study design and sampling

This research was conducted in Tehran, Iran, using a multi-stage cluster random sampling procedure involving 193 overweight or obese women. The participants had a body mass index (BMI) ranging from 25 to 40 kg/m^2^ and were aged between 18 and 48 years. The sample size formula, *N* = (([(*Z*1 − *α* + *Z*1 − *β*) × √1−*r*^2^]/*r*)2 + 2), *β* = 95%, and *α* = 0.05, *r* = 0.25, was used. The exclusion criteria were as follows: total daily energy intake outside of 800–4,200 kcal (17,556–3,344 kJ) (16), presence of metabolic diseases, menopause, pregnancy, breastfeeding, taking lipid-lowering and blood glucose-lowering agents, alcohol consumption, and smoking. All the participants of our study signed an informed consent form, and all the methods of our study were performed in accordance with the relevant guidelines and regulations.

### NOVA calculation

For evaluating the food consumption of participants throughout the previous year, we utilized a 147-item semi-quantitative food frequency questionnaire (FFQ) [its validity and reliability have already been authorized (17, 18)]. Participants evaluated their consumption frequency using four categories: daily, weekly, monthly, and infrequent. Using home measures, the portion sizes of the consumed foods were converted to grams (19). To evaluate energy intake and nutrients, version 7.0 of NUTRITIONIST IV was used. To evaluate NOVA components, we used the same method as our previous study (15).

### Assessment of outcome

To measure QoL, we utilized a self-administered SF-36 (short-form questionnaire), which comprises 36 questions. Thirty-five of these questions are compressed into eight multi-item scales, namely physical functioning (PF), role-physical (RP), role-emotional (RE), general health (GH), bodily pain (BP), vitality (VT), social functioning (SF), and mental health (MH) (20).

(1) The PF scale is a 10-question scale that assesses a person’s ability to deal with daily physical demands such as personal hygiene, flexibility, and walking. (2) The RP scale is a 4-item scale that assesses how much physical limitations hinder activity. (3) The BP scale is a 2-item measure that evaluates the discomfort felt in the last 4 weeks and how much that pain interfered with routine work duties. (4) The GH scale is a 5-item questionnaire that evaluates personal perceptions of general health. (5) The VT scale is a 4-item scale that evaluates a person’s sense of vigor, energy, and weariness. (6) The SF scale is a 2-item scale that assesses how long and how much physical health or emotional problems interfered with family, friends, and other social contacts in the previous 4 weeks. (7) The RE scale is a 3-item questionnaire that assesses how much emotional problems interfere with work or other activities. (8) The MH scale is a 5-item questionnaire to examine anxiety and depression symptoms (20, 21). The SF-36 also consists of a question about self-evaluating health changes over the last year, which is not part of the 8 categories or the total SF-36 score. The score of each of these 8 dimensions ranged from 0 to 100 (worst health to highest health).

### Assessment of covariates

The demographic and socioeconomic conditions were estimated using a questionnaire that has been used in recent studies (15). Anthropometric measures, including height (m), waist circumference (WC) (cm), and hip circumference (HC) (cm), were measured using standard protocols (22). The waist-to-hip ratio (WHR) was computed as WC (cm)/HC (cm). A body composition analyzer (BIA) (Inbody Co., Seoul, Korea) was used to assess the individuals’ weight, BMI, fat mass index (FMI), fat-free mass (FFM), and body fat percentage (%) according to a predetermined methodology (23). More than that, we used the validated International Physical Activity Questionnaire (IPAQ) to obtain the physical activity status of the participants. Metabolic equation hours per week (MET-hours/week) were calculated for each subject by trained professionals (16, 24).

### Data analysis

The participants were categorized into tertiles of UPF consumption in grams according to the NOVA score. A one-way analysis of variance (ANOVA) and *χ*^2^ tests were performed to compare the mean difference of quantitative and frequency of categorical variables across UPF tertiles, respectively. An analysis of covariance (ANCOVA) adjusted for potential confounders (age, BMI, energy intake, and physical activity) was performed. We used the Bonferroni *post-hoc* test to find the statistically significant difference among UPF tertiles. Linear regression was performed to evaluate the association of UPF consumption (independent variable) with QoL factors (dependent variable). Model 1 was adjusted for age, BMI, physical activity, and total energy intake, while model 2 was additionally adjusted for education status. This analysis was presented as the *β*-value and a confidence interval (CI) of 95%. SPSS v.26 software was utilized for statistical analysis.

## Results

### Distribution of socio-demographic and lifestyle markers of participants according to tertiles of UPFs

The analyzed sample was composed of 193 women with 36.37 (SD 8.05) years of age and a weight of 80.76 (SD 12.13) kg (Table 1). The mean BMI and FFM were 30.90 (SD 4.20) kg/m^2^ and 46.69 (SD 5.62) kg, respectively. Statistically significant differences were found in the height of women across tertiles of the NOVA score (*p* < 0.05). In addition, 45.6% of participants had a bachelor’s degree or higher. The percentages of low-income and high-income subjects were 20 and 33.3%, respectively. There were no other significant differences in socio-demographic and anthropometric variables among NOVA score tertiles.

**Table 1:** Distribution of socio-demographic and lifestyle parameters of participants, according to tertiles of UPFs consumption (*n* = 193).

Quantitative variables	T1	T2	T3	*p*-value	*p*-value^*^
	(<383.681)	(383.681–467.713)	(>467.713)		
Frequency (*N*)	70	67	53		
Age (year)	36.17 ± 7.45	37.91 ± 8.02	34.80 ± 8.95	0.11	0.13^a^
PA (MET-hour-week)	1179.15 ± 1284.31	848.39 ± 872.71	982.28 ± 915.77	0.23	0.35^b^
Anthropometrics					
Weight (kg)	82.67 ± 12.06	78.59 ± 9.38	80.86 ± 14.84	0.14	0.26^c^
Height (cm)	162.44 ± 5.69	159.82 ± 6.53	162.60 ± 5.87	0.01	0.27^c^
Body fat (%)	41.81 ± 5.19	41.54 ± 5.16	40.90 ± 6.48	0.67	0.45^c^
Fat-free mass (kg)	47.78 ± 5.66	45.70 ± 5.45	46.65 ± 5.81	0.09	0.19^c^
Waist-to-hip ratio	0.94 ± 0.05	0.92 ± 0.05	0.93 ± 0.05	0.30	0.73^c^
BMI (kg/m^2^)	31.33 ± 4.08	30.76 ± 3.65	30.50 ± 5.04	0.53	0.30^c^
FMI (kg/m^2^)	13.26 ± 3.21	12.95 ± 2.94	12.86 ± 3.86	0.77	0.59^c^
Categorical variable					
Income status *n* (%)				0.60	0.12
Weak	19 (38)	21 (42)	10 (20)		
Moderate	33 (37.5)	31 (35.2)	24 (27.3)		
High	16 (38.1)	12 (28.6)	14 (33.3)		

UPFs, ultra processed foods; BMI, body mass index; FMI, fat mass index; METs, metabolic equivalents; PA, physical activity; SD, standard deviation; Quantitative variables were showed by means ± SD, and qualitative variables were showed by number (percentage). *p*-values resulted from one-way ANOVA analysis and chi-2 test. *p*-value < 0.05 was considered significant. *p-values resulted from ANCOVA analysis and were adjusted for age, BMI, PA, and energy intake.

a Variables just adjusted for BMI, energy intake, PA.

b Variables just adjusted for BMI, energy intake, age.

c Variables just adjusted for PA, energy intake, age.

### Dietary intakes of participants according to UPF tertiles among Iranian women

As shown in Table 2, all NOVA score subgroups increased significantly across tertiles of UPFs score (*p* < 0.05). In addition, vegetable consumption was lower according to higher adherence to the UPF intake tertiles (*p* < 0.05). On average, the total fiber intake among the women in the study was 40.89 (19.26) g. This amount decreased significantly across the tertiles (*p* < 0.01). Foods/beverages containing EPA and DHA were consumed in a small amount. Overall, vitamins and minerals were not statistically different between the tertiles, even after adjusting for energy intakes.

**Table 2:** Dietary intakes of participants based on UPFs tertiles among Iranian women (*n* = 193).

Variables	Total	T1	T2	T3	*p*-value^*^
	Mean ± SD	(<383.681)	(383.681–467.713)	(>467.713)	
NOVA subgroups					
Nondairy beverages (g/d)	168.90 ± 88.20	120.36 ± 23.89	156.99 ± 32.10	251.18 ± 128.68	<0.001
Cookies-cakes (g/d)	96.47 ± 36.96	80.33 ± 26.64	96.98 ± 28.14	117.95 ± 47.43	<0.001
Dairy beverages (g/d)	45.91 ± 24.61	38.07 ± 18.33	46.45 ± 21.94	55.98 ± 31.24	<0.001
Potato chips-salty snack (g/d)	22.61 ± 14.79	16.90 ± 9.49	24.58 ± 11.24	27.85 ± 21.16	0.001
Processed meat-fast food (g/d)	38.71 ± 25.38	26.44 ± 11.47	38.29 ± 10.88	56.11 ± 39.39	<0.001
oil_Sause (g/d)	20.04 ± 10.38	18.53 ± 10.00	19.13 ± 8.94	23.31 ± 12.01	0.002
Sweet (g/d)	36.98 ± 20.42	33.43 ± 17.22	36.75 ± 18.08	42.16 ± 25.97	<0.001
Food groups					
Caffeine (mg/d)	150.15 ± 167.58	137.08 ± 103.89	151.24 ± 245.84	166.98 ± 97.26	0.69
Fruits (g/d)	543.58 ± 341.89	600.84 ± 337.97	504.36 ± 331.63	516.52 ± 356.34	0.54
Vegetables (g/d)	433.67 ± 264.77	499.84 ± 251.09	374.00 ± 198.64	421.23 ± 334.72	0.03
Whole grains (g/d)	7.08 ± 9.76	6.58 ± 8.12	7.22 ± 8.81	7.59 ± 12.73	0.82
Refined grains (g/d)	425.85 ± 210.39	472.78 ± 176.92	365.94 ± 177.06	440.12 ± 269.93	0.55
Macronutrients					
Protein (% of total energy)	14.00 ± 2.54	14.33 ± 2.70	13.46 ± 2.18	14.25 ± 2.68	0.54
Total fat (% of total energy)	32.21 ± 5.96	32.65 ± 6.53	31.34 ± 5.65	32.74 ± 5.51	0.98
Carbohydrate (% of total energy)	56.77 ± 6.28	55.81 ± 6.83	58.27 ± 5.78	56.12 ± 5.86	0.99
Total fiber (g/d)	44.90 ± 19.27	54.46 ± 18.67	36.81 ± 13.95	42.40 ± 20.70	<0.001
Fat subgroups					
SFA (g/d)	28.15 ± 11.62	31.09 ± 12.02	24.63 ± 9.94	28.72 ± 12.07	0.40
PUFA (g/d)	20.12 ± 9.78	22.75 ± 10.71	17.59 ± 9.82	19.83 ± 7.34	0.35
MUFA (g/d)	31.28 ± 12.80	34.72 ± 13.38	27.20 ± 11.69	31.92 ± 12.09	0.69
EPA (g/d)	0.03 ± 0.04	0.04 ± 0.04	0.03 ± 0.04	0.03 ± 0.04	0.44
DHA (g/d)	0.11 ± 0.13	0.10 ± 0.12	0.11 ± 0.12	0.11 ± 0.13	0.46
Trans fat (g/d)	0.001 ± 0.002	0.001 ± 0.002	0.001 ± 0.002	0.000 ± 0.001	0.57
Vitamins and minerals					
Vitamin D (μg)	1.99 ± 1.66	2.25 ± 2.05	1.75 ± 1.40	1.93 ± 1.30	0.80
Folate (μg/d)	597.45 ± 181.73	670.29 ± 141.47	525.20 ± 178.13	592.42 ± 198.90	0.67
Sodium (mg/d)	4245.93 ± 1503.84	4592.25 ± 1353.59	3859.28 ± 1390.00	4278.54 ± 1734.94	0.76
Potassium (mg/d)	4332.83 ± 1592.86	4879.20 ± 1367.59	3883.75 ± 1561.83	4172.89 ± 1724.52	0.19
Selenium (mg/d)	118.16 ± 42.71	129.13 ± 37.74	105.10 ± 42.04	120.27 ± 46.07	0.50

UPFs, ultra processed foods; SD, standard deviation; SFA, saturated fatty acids; PUFA, polyunsaturated fatty acids; MUFA, monounsaturated fatty acids; EPA, eicosapentaenoic acid; DHA, docosahexaenoic acid; *p*-values are resulted from ANOVA analysis. *p*-value < 0.05 was significant. *p-values presented resulted from ANCOVA analysis and were adjusted for energy.

### The subcategories of QoL differences among tertiles of UPFs

The mean score of the total QoL scale was 66.90 (SD 24.19) among Iranian women (Table 3). Regarding QoL subscales, role-physical and mental health domains were significantly different across tertiles of UPF intakes (*p* < 0.05). Women with lower adherence to UPF intake (first tertile) had a higher mean score in the role-physical domain, with a score of 86.03 (SD 34.39), compared to the third tertile. In addition, the first tertile of UPF consumption exhibited a higher mental health score of 79.38 (SD 20.77) compared to the third tertile of UPF consumption, which had a score of 70.82 (SD 21.29). These differences remained significant after adjusting confounders (*p* = 0.01 and *p* = 0.002). In addition, a marginal significance appeared in the vitality subgroup among tertiles of NOVA score in adjusted mode (*p* = 0.06).

**Table 3:** The subcategories of quality of life differences among tertiles of UPFs intakes (*n* = 193).

Variables^a^	Total	T1	T2	T3	*p*-value	*p*-value^*^
		(<383.681)	(383.681–467.713)	(>467.713)		
Quality of life	66.58 ± 24.34	66.01 ± 24.49	65.98 ± 26.44	68.14 ± 21.49	0.86	0.29
General health	65.79 ± 17.04	65.93 ± 18.21	64.96 ± 16.91	66.68 ± 15.80	0.86	0.78
Physical Functioning	81.94 ± 16.78	82.91 ± 18.15	79.04 ± 16.83	84.43 ± 14.39	0.19	0.17
Role-Physical	80.49 ± 39.40	86.03 ± 34.39^b^	83.33 ± 37.55	69.20 ± 46.11^c^	0.05	0.01
Role-Emotional	75.92 ± 42.70	78.95 ± 40.56	75.76 ± 43.18	72.00 ± 45.36	0.68	0.66
Social Functioning	71.68 ± 23.92	71.21 ± 25.28	72.50 ± 22.85	71.25 ± 23.85	0.94	0.66
Bodily Pain	62.05 ± 21.31	63.63 ± 18.72	60.10 ± 18.92	62.47 ± 27.09	0.62	0.71
Vitality	67.58 ± 19.04	69.93 ± 19.77	67.77 ± 17.78	64.13 ± 19.52	0.26	0.06
Mental Health	74.16 ± 22.85	79.38 ± 20.77^b^	71.31 ± 25.26^c^	70.82 ± 21.29^c^	0.05	0.002
Health Transition Item	45.52 ± 27.35	47.43 ± 28.37	40.53 ± 23.93	49.50 ± 29.66	0.16	0.19

*p One way ANCOVA. *p*-values < 0.05 were considered significant and between 0.05 to 0.07 were considered marginal significance. Variables are adjusted to energy intake, age, PA, BMI. Bonferroni post-hoc test was used.

a Mean ± SD was presented.

b Shows significant differences of variables between tertiles.

c Shows significant differences of variables between tertiles.

### Relationship between UPF intake and QoL and its subgroups

Table 4 shows the relationship between NOVA score tertiles, QoL score, and its eight scales. Tertile 1 was chosen as the reference group. Being in the third tertile of consumption of UPFs decreased by 16,829 times in the role-physical domain units (*β* = −16.82, 95% CI: −30.94–2.71, *p* = 0.01), regardless of confounding variables in the crude model. This significance was improved after adjusting for confounders in model 1 (*β* = −21.90, 95% CI: −36.18–7.62, *p* = 0.003). Finally, women in the third tertile of UPF intake had a 23.59 unit lower score in the limitation in physical capabilities and activity (score of role-physical) (*β* = −23.59, 95% CI: −37.77–9.40, *p* = 0.001). In addition, another subgroup of the QoL scale called vitality had a negative association with the third tertile of UPF intake in model 1 (*β* = −8.48, 95% CI: −16.18–0.78, *p* = 0.03). The highest adherence to UPF intake (tertile 3) reduced by 8.76 units in addressing feelings of energy and fatigue (vitality domain) in model 2 (*β* = −8.76, 95% CI: −16.42–1.11, *p* = 0.02). Moreover, the mental health of the participants was inversely related to the second (*β* = −8.06, 95% CI: −15.66–0.47, *p* = 0.03) and third (*β* = 8.55, 95% CI: −16.74–0.36, *p* = 0.04) tertiles of UPF consumption in the crude model. This significance became even stronger in models 1 and 2. Finally, 15.78 decreasing units were found at mental states of anxiety and depression (mental health scale) between participants of the third tertile of UPF intake (*β* = −15.78, 95% CI: −24.11–7.45, *p* < 0.001).

**Table 4:** Association between UPFs tertiles with quality of life and its subgroups in Iranian women (*n* = 193).

Variables	NOVA score	*β* ^*^	95% CI	*p*-value
Quality of life				
Crude				
	T2	−0.033	−8.15–8.09	0.99
	T3	2.12	−6.62–10.87	0.63
Model 1				
	T2	−3.86	−12.96–5.22	0.40
	T3	3.98	−5.56–13.53	0.11
Model 2				
	T2	−6.07	−14.71–2.56	0.06
	T3	0.01	−8.86–8.90	0.99
General health				
Crude				
	T2	−0.96	−6.71–4.78	0.74
	T3	0.75	−5.44–6.95	0.81
Model 1				
	T2	−1.98	−8.21–4.24	0.53
	T3	−1.99	−8.55–4.56	0.55
Model 2				
	T2	−2.04	−8.27–4.18	0.52
	T3	−2.06	−8.62–4.49	0.53
Physical Functioning				
Crude				
	T2	−3.86	−9.48–1.75	0.17
	T3	1.51	−4.54–7.57	0.62
Model 1				
	T2	−5.15	−10.52–0.22	0.06
	T3	−1.27	−6.83–4.27	0.65
Model 2				
	T2	−4.84	−10.24–0.54	0.07
	T3	−0.98	−6.55–4.59	0.73
Role-Physical				
Crude				
	T2	−2.69	−15.79–10.39	0.68
	T3	−16.82	−30.94–2.71	0.01
Model 1				
	T2	−2.91	−16.73–10.90	0.67
	T3	−21.90	−36.18–7.62	0.003
Model 2				
	T2	−4.64	−18.37–9.09	0.50
	T3	−23.59	−37.77–9.40	0.001
Role-Emotional				
Crude				
	T2	−3.19	−17.58–11.19	0.66
	T3	−6.95	−22.46–8.56	0.38
Model 1				
	T2	−7.24	−23.97–9.47	0.39
	T3	−6.56	−24.16–11.04	0.46
Model 2				
	T2	−7.28	−24.01–9.45	0.39
	T3	−6.60	−24.22–11.02	0.46
Social Functioning				
Crude				
	T2	1.28	−6.79–9.36	0.75
	T3	0.03	−8.67–8.74	0.99
Model 1				
	T2	1.67	−6.92–10.27	0.70
	T3	−2.63	−11.68–6.41	0.56
Model 2				
	T2	1.40	−7.12–9.93	0.74
	T3	−2.98	−11.97–5.99	0.51
Bodily Pain				
Crude				
	T2	−3.52	−10.70–3.65	0.33
	T3	−1.16	−8.90–6.58	0.76
Model 1				
	T2	−2.46	−10.60–5.68	0.55
	T3	−3.50	−12.07–5.06	0.42
Model 2				
	T2	−2.30	−10.42–5.82	0.57
	T3	−3.30	−11.85–5.24	0.44
Vitality				
Crude				
	T2	−2.15	−8.53–4.23	0.50
	T3	−5.80	−12.68–1.08	0.09
Model 1				
	T2	−3.43	−10.75–3.87	0.35
	T3	−8.48	−16.18–0.78	0.03
Model 2				
	T2	−3.65	−10.92–3.60	0.32
	T3	−8.76	−16.42–1.11	0.02
Mental Health				
Crude				
	T2	−8.06	−15.66–0.47	0.03
	T3	−8.55	−16.74–0.36	0.04
Model 1				
	T2	−10.39	−18.33–2.46	0.01
	T3	−15.57	−23.92–7.22	<0.001
Model 2				
	T2	−10.55	−18.47–2.64	0.009
	T3	−15.78	−24.11–7.45	<0.001
Health Transition Item				
Crude				
	T2	−6.89	−16.04–2.25	0.14
	T3	2.07	−7.78–11.93	0.68
Model 1				
	T2	−7.28	−17.33–2.76	0.15
	T3	2.30	−8.27–12.87	0.67
Model 2				
	T2	−7.70	−17.60–2.20	0.07
	T3	1.75	−8.67–12.18	0.74

UPFs, ultra processed foods. All values are presented as 95% Confidence intervals (CI). *p*-value < 0.05 were considered significant. *p*-values between 0.05 to 0.07 were considered marginal significance. β* regression coefficients refer to the UPF tertiles relationship. Model 1: Adjusted for age, energy intake, BMI and physical activity. Model 2: Adjusted for model 1 + education status.

## Discussion

The current study investigated the associations between UPF intake and QoL in Iranian women for the first time. Participants with higher UPF consumption displayed a lower QoL (after adjustment for possible confounders). In other words, we observed a significant negative association between UPF intake and role-physical, mental health, and vitality in both models (crude and adjusted).

Similar to our study, another study in 2020 indicated that individuals with unhealthy dietary patterns, such as a Western diet characterized by high consumption of refined grains, red or processed meat, and sugary carbonated beverages, had lower QoL scores (25). Another study showed that adherence to healthy dietary patterns, such as the Mediterranean diet, could benefit at least one of the QoL domains (9). A study conducted in Paraguay in 2022 demonstrated that consumption of foods rich in sodium, free sugars, fat, and nitrites was associated with a lower QoL and insufficient sleep duration (26), and a study conducted in Brazil in 2020 revealed that a decrease in the consumption of UPFs led to a reduction in the range of body mass index and waist circumference and an improvement in QoL (27). Magaly Aceves et al. showed that often, due to the cheaper, more convenient, and lower nutritional quality of highly processed foods and their potential health risks, it can lead to increased medical costs and decreased productivity and quality of life (28). In addition, studies showed that increasing consumption of processed foods can affect self-confidence and social interactions by affecting physical appearance, such as weight gain (29–33).

Numerous situations in life, such as living arrangements, migration, loss of loved ones, being unemployed, aging, and a lack of social relationships, could affect food intake and nutritional status and, as a result, affect the QoL in individuals (34–36). On the other hand, nowadays, people are under tremendous financial pressure, especially in low-income countries such as Iran, and they have to work round the clock to satisfy their basic needs. Hence, they are too tired to spend time cooking healthy meals at home, and instead, they prefer consuming ready-to-eat foods or purchasing food from takeaway outlets. UPFs can have so many detrimental effects on various aspects of human health. Cheng et al. observed that higher intake of UPFs is associated with a greater risk of overall cancer, as well as an increased risk of overall mortality (37). A diet high in UPF content is nutritionally inferior, as it tends to be higher in energy, saturated fats, salt, and free sugars, while being lower in several micronutrients and fiber (38). Furthermore, evidence has shown the strong obesity-promoting potential of UPFs (39), which is a risk factor for several metabolic diseases, including some cancers in women (40–42).

Inflammation is a key mediator in chronic diseases such as diabetes and depression (43–47). Ultra-processed foods can induce metabolic endotoxemia, increase inflammatory cytokines, and impair endothelial function (48, 49). Furthermore, highly processed foods can alter neurotransmissions by altering the availability and activity of neurotransmitters such as dopamine, serotonin, and glutamate (50). These foods can stimulate the reward pathway, increase food cravings, and create addictive eating behaviors (51, 52). Studies have linked the consumption of highly processed foods with lower levels of dopamine and serotonin in the brain and higher cases of food addiction, overeating, and depression (53, 54). It also indicated that UPFs can affect the gut microbiome, potentially leading to chronic inflammation and diseases, such as inflammatory bowel disease (IBD), irritable bowel syndrome (IBS), and colorectal cancer (55).

Emerging evidence has shown additional properties of UPFs that could contribute to adverse human health outcomes. These include the use of controversial food additives, the formation of new contaminants during ultra-processing, and the migration of toxic contaminants from food packaging (37). Bisphenols and phthalates are endocrine-disrupting chemicals usually found in food storage, packaging, and contact materials, and higher urinary concentrations of phthalates and bisphenols have been found in people with higher UPF consumption (56). The available data on bisphenols have consistently shown many toxic effects, including DNA damage and impacts on the nervous and immune systems (57).

The present study has some strengths. To the best of our knowledge, this is the first study to investigate the associations between UPF consumption and QoL among obese and overweight Iranian women. Furthermore, dietary intake was assessed utilizing a validated questionnaire. Nevertheless, the study had several limitations. It was observational; hence, causal inference is limited. In addition, some errors may be present in the dietary assessment due to recall bias and misclassification errors. Moreover, our results are not generalizable to men and normal-weight women.

We observed an inverse association between UPF consumption and role-physical, mental health, and vitality in overweight and obese women. The causality may not be implied because of the observational nature of the study, but these findings highlight the importance of considering UPF intake in diets. Our findings suggest that limiting UPF intake could be beneficial in preventing/reducing some diseases and improving the QoL in Iranian women.

## Conclusion

In conclusion, this study has highlighted the significant association between UPF consumption and QoL among overweight and obese women. The findings indicate that higher UPF intake is associated with poorer QoL. To gain a better understanding of the relationship between UPF and quality of life, we suggest conducting a series of clinical studies so that we can determine the cause-and-effect relationships and make practical recommendations for the future.

## Data availbility statement

The raw data supporting the conclusions of this article will be made available by the authors, upon request to the corresponding author.

## Ethics statement

The studies involving humans were approved by Department of Community Nutrition, School of Nutritional Sciences and Dietetics, Tehran University of Medical Sciences (TUMS), Tehran, Iran. The studies were conducted in accordance with the local legislation and institutional requirements. The participants provided their written informed consent to participate in this study.

## Author contributions

DH: Writing – original draft, Writing – review & editing. FS: Formal analysis, Writing – review & editing. NB: Writing – review & editing. AS: Writing – review & editing. MH: Writing – review & editing. JB: Writing – review & editing. KM: Writing – review & editing.

## Glossary

QoL: Quality of life
CVD: Cardiovascular disease
WHO: World Health Organization
FFQ: Food frequency questionnaire
BMI: Body mass index
SD: Standard deviation
UPF: Ultra-processed food
WHR: Waist-to-hip ratio
HC: Hip circumference
WC: Waist circumference
PF: Physical functioning
RP: Role-physical
BP: Bodily pain
GH: General health
VT: Vitality
MH: Mental health
SF: Social functioning
RE: Role-emotional
FMI: Fat mass index
FFM: Fat-free mass
BIA: Bioimpedance analysis
IPAQ: International physical activity questionnaire
BF: Body fat
ANOVA: Analysis of variance
ANCOVA: Analysis of covariance
CI: Confidence interval
IBD: Irritable bowel syndrome
IBS: Inflammatory bowel disease
